# Diagnostic Uptake of Targeted Sequencing in Adults With Steatotic Liver Disease and a Suspected Genetic Contribution

**DOI:** 10.1111/liv.70010

**Published:** 2025-02-13

**Authors:** Luisa Ronzoni, Serena Pelusi, Vittoria Moretti, Francesco Malvestiti, Hadi Eidgah Torghabehei, Oveis Jamialahmadi, Jessica Rondena, Cristiana Bianco, Giulia Periti, Maria Rosaria De Filippo, Stefano Romeo, Daniele Prati, Luca Valenti

**Affiliations:** ^1^ Precisione Medicine Lab, Biological Resource Center and Department of Transfusion Medicine Fondazione IRCCS ca' Granda Ospedale Maggiore Policlinico Milano Milan Italy; ^2^ Department of Pathophysiology and Transplantation Università Degli Studi di Milano Milan Italy; ^3^ Omic Sciences Lab, Scientific Direction Fondazione IRCCS ca' Granda Ospedale Maggiore Policlinico Milano Milan Italy; ^4^ Department of Molecular and Clinical Medicine, Institute of Medicine, Academy, Wallenberg Laboratory University of Gothenburg Gothenburg Sweden; ^5^ Department of Cardiology Sahlgrenska University Hospital Gothenburg Sweden; ^6^ Clinical Nutrition Unit, Department of Medical and Surgical Sciences University Magna Graecia Catanzaro Italy

**Keywords:** diagnostic uptake, next‐generation sequencing, polygenic risk score, steatotic liver disease, targeted sequencing

## Abstract

**Background and Aims:**

In patients with steatotic liver diseases (SLD), genetic factors may account for severe liver involvement despite mild or absence of triggering factors or a strong family history. Aim of this study was to examine the diagnostic uptake of targeted sequencing (TS), covering both coding and non‐coding regions, of a broad panel of 82 liver and lipid metabolism genes in patients with unexplained SLD.

**Methods:**

We enrolled 49 adult patients with SLD and a suspected genetic contribution. Genetic variants were detected through a customised TS panel, whereas the contribution of common genetic variation to the individual susceptibility to SLD was captured by a polygenic risk score (SLD‐PRS).

**Results:**

A diagnosis of rare Mendelian disorder was established in 11 patients (22%), independently of age or family history. Rare variants possibly contributing to clinical phenotype were detected in additional 29 patients (59%). Increased SLD‐PRS values were detected in 17 patients (35%), enabling an increase in diagnostic uptake of 24%, especially in those without a strong family history (*p* = 0.03). Genetic diagnosis allowed refinement of clinical management in 23 (47%) patients.

**Conclusions:**

The diagnostic uptake of TS was 22% for Mendelian disorder and 59% for possible contribution to clinical phenotype in selected adult patients with SLD. Evaluation of common variants, as captured by SLD‐PRS, yields complementary information increasing the overall utility of the genetic examination.

**Abbreviations**
ALT: alanine aminotransferase
AST: aspartate transaminase
BMI: body mass index
CNV: copy number variants
GGT: gamma‐glutamyl transferase
HCC: hepatocellular carcinoma
LDL: low density lipoprotein
LP: likely pathogenic
LSM: liver stiffness measurement
MAF: minor allele frequency
NGS: next‐generation sequencing
P: pathogenic
PRS: polygenic risk score
SLD: steatotic liver disease
SLD‐PRS: polygenic risk score for steatotic liver disease
T2D: type‐2 diabetes
TS: customised targeted gene panel sequencing
VCTE: vibration controlled transient elastography
VUS: variant of unknown significance
WES: whole‐exome sequencing

**Summary**

- In a clinically relevant fraction of patients with steatotic liver diseases (SLD), the development and progression of liver involvement remains unexplained despite extensive clinical, laboratory and imaging workup.
- Next‐generation targeted sequencing, with complementary evaluation of rare variants and of genetic predisposition to SLD due to common genetic variation, allowed refinement of clinical management in 47% of adult patients with SLD with a suspected genetic contribution.
- Genetic testing can be included in the diagnostic workup in specific subgroups of patients with SLD indistinguishable through conventional diagnostic approaches.

## 1 Introduction

Steatotic liver disease (SLD) affects around 30% of the global population and has become the leading cause of cirrhosis that is globally the eleventh cause of death [1, 2]. An early diagnosis is essential to promote targeted management to halt progression of liver disease and improve outcomes [3, 4]. The recent SLD nomenclature change distinguishes metabolic dysfunction associated SLD (MASLD) from SLD associated with monogenic disorders and cryptogenic SLD, with implications for the prognosis and disease management [1]. However, despite about 10%–15% of patients develop SLD in the absence of overweight, [5], potentially bearing a worse prognosis, [6] and a strong family history in first‐degree relatives is associated with a high risk of developing SLD progressing to advanced fibrosis [7], the genetic contribution to SLD is still rarely evaluated in clinical practice. Indeed, up to 30% of individuals with cirrhosis and up to 14% of adult patients waiting for liver transplantation remained undiagnosed [8], or diagnosed as MASLD thanks to the unspecific presence of a single metabolic risk factor. The implementation of next‐generation sequencing (NGS) in diagnostic work‐up of adult patients with SLD could lessen the burden on patients with unexplained liver disease [9, 10]. During the last years, pilot studies in small cohorts of adults with SLD have demonstrated the utility of genomic analysis in providing a diagnosis in patients with cryptogenic liver disorders as well as in contributing to explain disease pathogenesis [11, 12, 13, 14, 15, 16].

Despite these promising results, NGS is still only available at selected tertiary centres, and it is not yet routinely used in clinical practice, in particular of adults [17, 18]. Alternative NGS approaches are whole‐exome sequencing (WES), covering the protein‐coding regions of almost all genes, and targeted sequencing (TS), focused on a set of genes specific for liver disease. Advantages of TS included a higher mean coverage depth, and the possibility to evaluate non‐coding regions (e.g., gene promoters, 5′/3′‐untranslated regions with regulatory function), leading to an increase in analytical sensitivity extending to the possible detection of mosaicism. Furthermore, TS findings can be interpreted more easily and avoid incidental secondary findings [19].

Besides identifying rare variants causative of Mendelian diseases, NGS allows to evaluate the contribution of common risk variants. We previously developed a polygenic risk score for SLD (SLD‐PRS, previously referred to as PRS‐5), based on the combination of common variants in five genes (*PNPLA3*, *TM6SF2*, *GCKR*, *MBOAT7* and *HSD17B13*), capturing the genetic predisposition to develop a form of SLD at higher risk of hepatic complications, including cirrhosis, hepatocellular carcinoma (HCC) and liver events [20]. We proposed the utility of the SLD‐PRS in the diagnosis of patients with SLD as a complementary approach to rare genetic variants detection [15].

Within this context, the aim of this study was to examine the diagnostic uptake of the customised validated TS panel, including 82 liver and lipid metabolism‐related genes, in a cohort of unrelated adult patients with SLD with a suspected genetic component, and as a complementary approach, we assessed SLD‐PRS to probe the contribution of common genetic variation to this phenotype.

## 2 Methods

### 2.1 Study Cohort

Consecutive adult patients evaluated at the Hepatology outpatient service of the Fondazione IRCCS Cà Granda Ospedale Maggiore Policlinico of Milan due to the presence of SLD with suspected genetic contribution were included in the study. SLD was defined as the presence of hepatic steatosis as detected by abdominal ultrasound and/or controlled attenuation parameter (CAP) ≥ 275 dB/m at vibration controlled transient elastography (VCTE) with Fibroscan and/or liver histology. We ruled out triggering factors other than metabolic alterations, including (a) daily alcohol intake ≥ 30/20 g/day in males/females; (b) concurrent known liver diseases, including chronic viral and autoimmune hepatitis or celiac disease; (c) other already diagnosed genetic liver diseases such as hemochromatosis, Wilson's disease, alpha1‐antitrypsin deficiency, hypobetalipoproteinemia (HBL); and (d) steatogenic/hepatotoxic drugs. In addition, to identify people with a higher likelihood of having a large genetic contribution to SLD, we required the presence of at least one amongst the following criteria: (a) absence of overweight (body mass index (BMI) < 25/23 kg/m^2^ for Europeans and Asians, respectively); (b) presence of a positive family history for advanced liver fibrosis or HCC in a first degree relative; (c) an age at onset < 25 years unrelated to obesity; (d) otherwise unexplained extra‐hepatic involvement suggesting a systemic disorder. To better characterise SLD subphenotypes, we determined the concomitant presence of hyperferritinemia or dyslipidemia. Hyperferritinemia was defined as the presence of ferritin levels > 200 μg/L in women and > 300 μg/L in men, associated with either increased transferrin saturation (> 45%) or evidence of excessive liver iron accumulation by histology or nuclear magnetic resonance (NMR T2* measurement < 8 ms) or as isolated hyperferritinemia > 800 μg/L in the absence of elevation of inflammatory markers [21]. Dyslipidemia encompassed HBL with decreased plasma lipid levels (total cholesterol < 115 mg/dL, low density lipoprotein—LDL cholesterol < 50 mg/dL, triglycerides < 45 mg/dL, or ApoB levels < 0.5 g/L), or hypercholesterolemia with persistent increase of LDL levels ≥ 190 mg/dL (see Data S1: Methods for further information on data collection).

The study protocol was approved by the Ethical Committee of Fondazione IRCCS Ca′ Granda (CE 125_2018bis). Written informed consent was obtained from each patient.

### 2.2 Targeted Sequencing

TS analysis was performed using a comprehensive liver disease targeted NGS validated panel including 82 liver‐related genes, classified into six categories: iron overload, lipid metabolism, cholestatic diseases, storage diseases, specific hereditary liver diseases and genes associated with susceptibility to liver diseases, as previously reported and detailed in the Data S1: Methods [19].

### 2.3 Variants Analysis and Interpretation

Variant prioritisation was performed as previously described [19]. Briefly, exonic non‐synonymous (missense, frameshift, nonsense), untranslated regions (UTR) or splice‐site variants were considered. Amongst these, we selected: (a) rare or low‐frequency variants with minor allele frequency (MAF) ≤ 0.05 according to Genome Aggregation Database (gnomAD); missense variants with MAF between 0.005 and 0.05 were considered only if CADD (Combined Annotation Dependent Depletion) score was ≥ 20; (b) variants reported as “pathogenic” or “likely pathogenic” according to ClinVar, as annotated by wANNOVAR, independent of MAF. We considered specific gnomAD subpopulation database according to individuals' ethnicity (gnomAD_Non‐Finnish European for individuals of South‐European ancestry).

**FIGURE 1:**
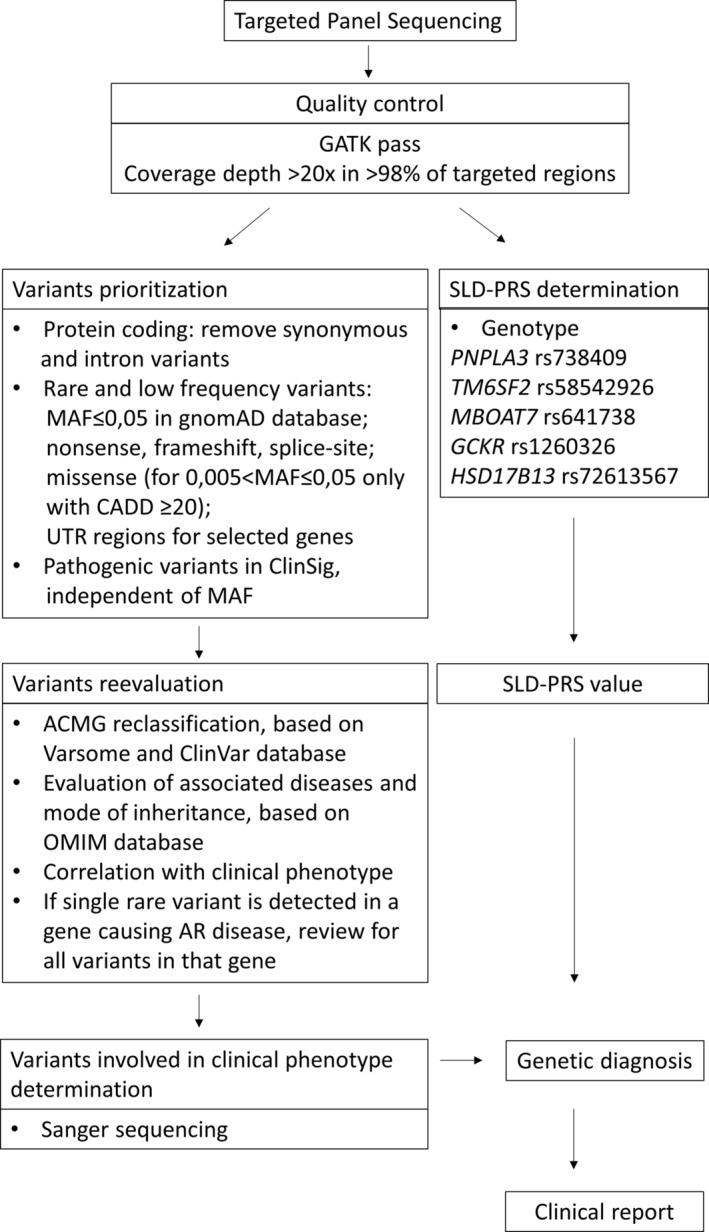
Diagnostic flow chart of variant filtering strategy, SLD‐PRS determination and genetic diagnosis reporting. ACMG, American College of Medical Genetics; AR, Autosomal recessive; MAF, Minor allele frequency; OMIM, Online Mendelian Inheritance in Man; PRS, Polygenic risk score; SLD, Steatotic liver disease.

All prioritised variants were then individually and visually re‐evaluated and classified according to the American College of Medical Genetics (ACMG) guidelines as follows: pathogenic (P), likely pathogenic (LP), unknown significance (VUS), likely benign (LB) and benign (B) [22] through the VarSome platform and NCBI ClinVar database (accessed on August 2024). The associated diseases and inheritance mode were considered, employing different filters for recessive or dominant disorders (MAF ≤ 0.01 or ≤ 0.001, respectively). When a single rare variant was detected in a gene causing an autosomal recessive disease, all variants in that gene were reviewed independent of MAF. Finally, molecular data were compared with clinical findings to detect genotype–phenotype concordance. Variants involved or likely involved in clinical phenotype determination were confirmed by Sanger sequencing, as described in Data S1: Methods. The pipeline for variants prioritisation and evaluation is reported in Figure 1.

### 2.4 SLD Polygenic Risk Score Determination

The genotypes for rs738409 (*PNPLA3* I148M), rs58542926 (*TM6SF2* E167K), rs641738 (*MBOAT7* C>T), rs1260326 (*GCKR* P446L) and rs72613567 (*HSD17B13* T>TA) were extracted for each patient from the “vcf” files. The SLD‐PRS was calculated as previously reported for the PRS‐5 [20] (Figure 1).

### 2.5 UK Biobank Validation

Candidate variants possibly contributing to clinical phenotype were searched for in exome sequencing data of unrelated Europeans participants the UK Biobank, and their impact on liver phenotypes assessed as described [23].

### 2.6 Genetic Diagnosis Determination

The following diagnostic outcomes were defined: (a) definite genetic diagnosis: when biallelic P/LP variants for autosomal recessive (AR) diseases or monoallelic P/LP variants for autosomal dominant (AD) or X‐linked disorders consistent with phenotype were detected; (b) suggested genetic diagnosis: when rare VUS consistent with phenotype and mode of inheritance were identified (c) possible genetic contribution to the clinical phenotype, when VUS in genes involved in disease pathogenesis were detected; (d) genetic predisposition to SLD due to common variants, when the SLD‐PRS was ≥ 0.495, in the absence of criteria a–c; (e) negative analysis, when no variants consistent with phenotype nor high SLD‐PRS value were identified.

The clinically relevant data generated (variants detected, SLD‐PRS genotypes and value, and the final genetic diagnosis) were next included in a clinical report, automatically generated using R software, as detailed in Data S1: Methods and Figure S1. The R code for implementing the automated pipeline is rendered freely available as Data S1: File.

### 2.7 Statistical Analysis

For descriptive statistics, categorical variables were shown as number and proportion and continuous variables as median and interquartile range (IQR). Observational associations were performed by fitting data to generalised linear models; logistic models were fit to examine binary traits. In multivariable models, analyses were adjusted for relevant covariates, as detailed in the Results section. Statistical analysis was carried out using the JMP Pro 16.0 Statistical Analysis Software (SAS Institute, Cary, NC). *p* values < 0.05 (two‐tailed) were considered statistically significant.

## 3 Results

### 3.1 Study Cohort

Forty‐nine unrelated patients were included in the study. Detailed demographic and clinical characteristics of the overall cohort and after stratification for the concomitant presence of hyperferritinemia or dyslipidemia are reported in Table 1 and in more details in Table S1. The clinical features of the two dyslipidemia subphenotypes are reported in Table S2. Thirty‐six individuals were males (73%), the majority (44; 90%) of Southern‐European ancestry and the median age at enrolment was 55 years (range: 20–80 years). After stratification for the concomitant presence of hyperferritinemia or dyslipidemia, patients with isolated SLD were older (*p* = 0.005), had higher values of liver enzymes and more severe fibrosis, as detected by LSM ≥ 8 kPa (*p* = 0.02); a lower proportion of these patients reported a positive family history (*p* = 0.01).

**TABLE 1:** Clinical features of the overall cohort and after stratification for concomitant presence of hyperferritinemia and dyslipidemia.

	Total cohort (*n* = 49)	SLD (*n* = 31)	SLD & hyperferritinemia (*n* = 12)	SLD & dyslipidemia (*n* = 6)	*p*
Age (years)	55 [41;61]	59 [45;62]	50 [38;55]	34 [27;45]	0.005
Sex, M	36 (73)	19 (61)	11 (92)	6 (100)	0.04
Caucasian, yes	44 (90)	28 (90)	11 (92)	5 (83)	0.8
Family history, yes	27 (55)	12 (39)	10 (83)	5 (83)	0.01
BMI (kg/m^2^)	27 [24;29]	27 [25;32]	27 [26;28]	26 [25;29]	0.7
T2D, yes	12 (24)	12 (39)	0	0	0.009
Liver fibrosis, yes	24 (49)	22 (71)	1 (8)	1 (17)	0.0001
Cirrhosis, yes	15 (31)	14 (45)	1 (8)	0	0.02
LSM (Kpa)	6.6 [4.5;19]	10 [6;22.5]	4.5 [3.5;6]	6 [4.5;7.5]	0.002
AST (IU/L)	35.5 [24;55]	44 [34;64]	25 [22;34]	25 [19;35]	0.005
ALT (IU/L)	44 [28;72]	47 [33;94]	30 [23;45]	28 [24;57]	0.02
GGT (IU/L)	50 [32;99]	68 [44;120]	32 [21;55]	30 [16;54]	0.008
LDL (mg/dL)	113 [84;143]	112 [86;128]	136 [94;143]	129 [22;248]	0.89
Triglycerides (mg/dL)	125.5 [98;187]	140 [103;204]	116 [101;174]	84 [37;137]	0.12
Ferritin (ng/mL)	256 [76;607]	187 [69;318]	936 [644;1098]	61 [47;301]	< 0.0001
Transferrin sat. (%)	na	na	44 [34:64]	na	

*Note:* Data are shown as *N* (%), or median [IQR], when appropriate. *p* values were calculated among pairs through Kruskal‐Wallis test for continuous variables (non‐normality assumed) and Fisher test for categorical variables.

Abbreviations: ALT, alanine aminotransferase; AST, aspartate transaminase; BMI, body mass index; GGT, gamma‐glutamyl transferase; LDL, low density lipoprotein; LSM, liver stiffness measurement; M, male; na, not available; T2D, type 2 diabetes.

### 3.2 Rare Variants Detection

In the overall cohort, 61 clinically relevant variants were detected in 49 patients (Table 2 and Table S3). In the majority of cases, patients harboured biallelic or monoallelic variants in only one gene (26 cases, 53%), whereas in 14 cases (29%) they harboured variants in more than one gene; in 5 of them, a rare variant was associated with a low frequency one. No clinically relevant variants were detected in 9 patients (18%).

**TABLE 2:** Demographics, clinical and genetic information of patients for whom a genetic diagnosis was established.

ID	Clinical phenotype (subgroup)	Age (y)	Sex	Gene	rsID	Variant (transcript, nucleotide and protein change)	gnomAD NFE	CADD score	Zygosity	ACMG	SLD‐PRS (NV < 0.495)	*PNPLA3* rs738409 C>G	Diagnosis (# OMIM, inheritance)
1	SLD	59	M	*ALDOB*	rs1800546	NM_000035: c.448G>C: p.A150P	0.005	31	Homo	P	0.5	GG	Hereditary Fructose Intolerance
													(# 229600, AR)
2	SLD	37	M	*LIPA*	rs116928232	NM_001127605: c.894G>A:	0.001	/	Homo	P	0.52	CG	Cholesteryl ester storage disease (CESD)
						p.Ser275_Gln298del							(# 278000, AR)
				*HFE*	rs1800562	NM_000410: c.845G>A: p.C282Y	0.07	25.7	Het	P			*HFE*‐related hemochromatosis
				*HFE*	rs1799945	NM_000410: c.187C>G: p.H63D	0.15	24.4	Het	P			(# 235200, AR)
3	SLD	59	F	*LPL*	rs191402029	NM_000237: c.542G>A: p.G181D	0.000003	27	Het	P	0.13	CC	Combined hyperlipidemia (# 144250, AD)
				*ABCB4*	rs375315619	NM_000443: c.1529A>G: p.N510S	0.0001	23.5	Het	VUS			
				*ABCB4*	rs8187788	NM_000443: c.217C>G: p.L73V	0.001	5.2	Het	VUS			
4	SLD	60	M	*JAG1*	rs761187116	NM_000214: c.3083 T>C: p.I1028T	0.000004	24.2	Het	P	0.53	CC	Alagille syndrome
													(# 118450, AD)
5	SLD	57	M	*TERT*	rs34094720	NM_001193376: c.1234C>A:	0.005	4.9	Het	VUS	0.73	CG	Suggested *TERT*‐associated telomerase
						p.H412N							Syndrome (# 614742, AD)
6	SLD	64	M	*RTEL*	rs748756306	NM_001283009: c.3051C>A:	0.00003	23.3	Het	VUS	0.16	CC	Suggested *RTEL1*‐associated telomerase
						p.D1017E							Syndrome (# 616373, AD)
7	SLD &	23	M	*HFE*	rs1800562	NM_000410: c.845G>A: p.C282Y	0.07	25.7	Het	P	0.29	CC	*HFE*‐related hemochromatosis
	Hyperferritinemia			*HFE*	rs1799945	NM_000410: c.187C>G: p.H63D	0.15	24.4	Het	P			(# 235200, AR)
8	SLD &	49	M	*HFE*	rs1800562	NM_000410: c.845G>A: p.C282Y	0.07	25.7	Homo	P	0.28	CG	*HFE*‐related hemochromatosis
	Hyperferritinemia			*TFR2*	rs41295942	NM_003227: c.2255G>A: p.R752H	0.03	33	Het	LB			(# 235200, AR)
				*HJV*	rs864622168	NM_213653: c.187_188insGAG:	0.0006	/	Het	LB			
						p.G69_R70insG							
9	SLD & Dyslipidemia	49	M	*LDLR*	rs745343524	NM_000527: c.1301C>T: p.T434M	0^a^	15.5	Het	P	0.19	CC	Familial Hypercholesterolemia type 1
	(hypercholesterolemia)												(# 143890, AD)
10	SLD & Dyslipidemia (HBL)	20	M	*APOB*	rs777287390	NM_000384: c.7286G>T: p.S2429X	0	40	Het	P	0.73	CG	Heterozygous Familial HBL (# 615558, AR)
11	SLD & Dyslipidemia (HBL)	29	M	*APOB*	na	NM_000384: c.3696 + 2 T>G	na	24.6	Het	LP	0.06	CC	Heterozygous Familial HBL (# 615558, AR)

Abbreviations: F, female; Het, heterozygous; Homo, homozygous; LB, likely benign; LP, likely pathogenic; M, male; na, not available; P, pathogenic; SLD, steatotic liver disease; VUS, variant unknown significance.

^a^ Allele frequency in African ancestry.

**FIGURE 2:**
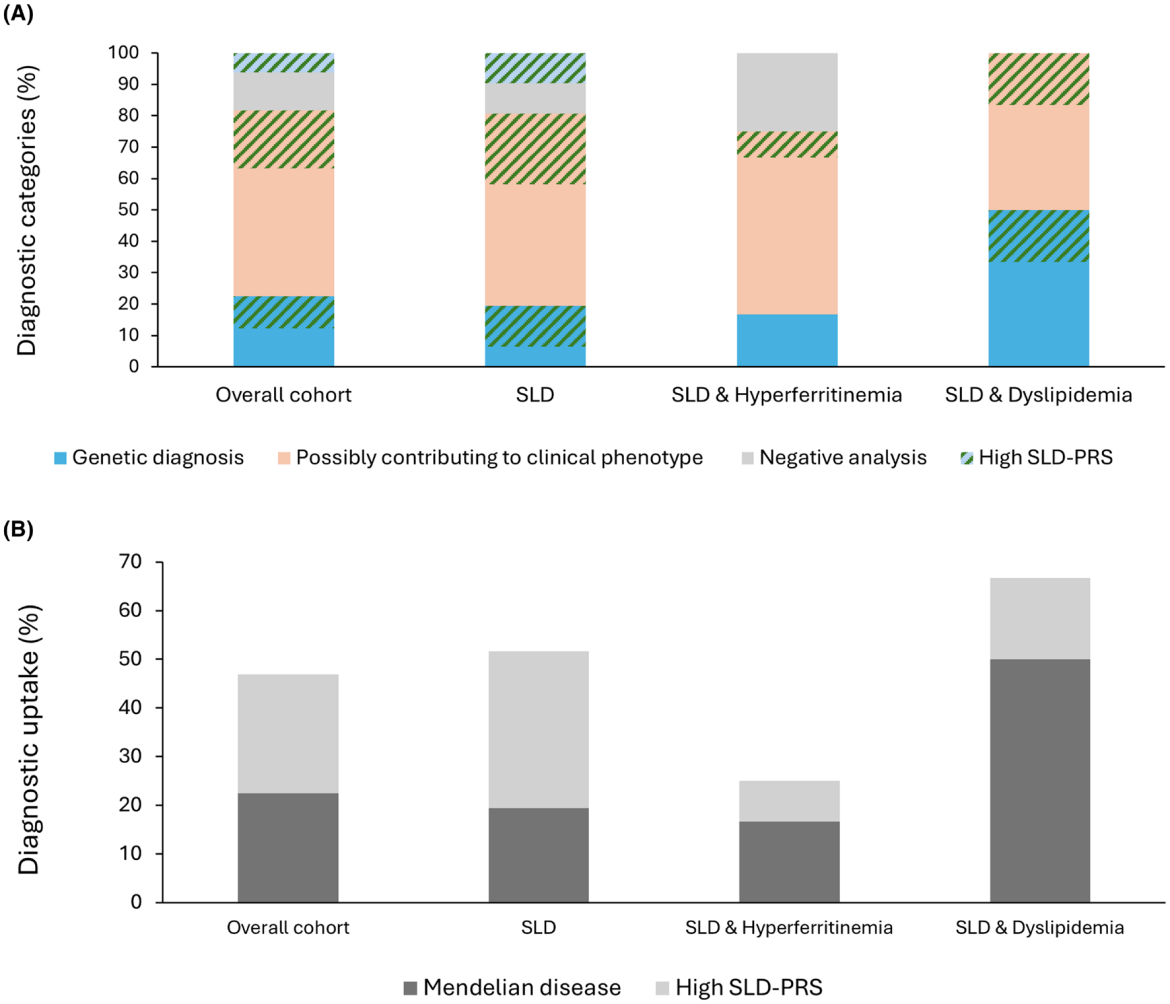
(A) Frequency of the diagnostic categories, based on rare high functional impact variants, and distribution of high SLD‐PRS values in the overall cohort and in the clinical phenotypes. (B) Diagnostic uptake in the overall cohort and in the clinical phenotypes and impact of high SLD‐PRS.

Combining clinical and molecular data, we established a clinical diagnosis of a Mendelian disorder, definite or suggested, in 11 patients (22%). Rare variants possibly contributing to clinical phenotype were detected in additional 29 patients (59%). When we stratified according to the concomitant presence of hyperferritinemia or dyslipidemia, a nominally higher diagnostic uptake was obtained in patients with dyslipidemia, although the difference was not statistically significant (*p* = 0.2; Figure 2A).

To further investigate the role of rare variants possibly contributing to the clinical phenotype, whose clinical significance remained uncertain, we tested the association of these rare variants with liver and metabolic traits using the exome sequencing data from the UKBB. We found that for 14 variants there were carriers in UKBB to allow for association testing. Three individual variants showed a significant association with liver traits: besides the low‐frequency *SERPINA1* rs28929474 (PiZ allele), a main genetic determinant of liver disease, [24] we identified *ABCB4* rs375315619 and *APOB* rs12691202, supporting their role in phenotype determination (Table S4 and Data S1: Results).

### 3.3 Genetic Diagnosis of a Mendelian Disorder

We established a genetic diagnosis of Mendelian disorder in six patients with SLD alone; in three of them, a genetic disease usually manifesting in the paediatric age, and therefore poorly investigated in adulthood, was detected: specifically, Alagille syndrome (OMIM #118450), cholesteryl ester storage disease (CESD; OMIM #278000) and hereditary fructose intolerance (OMIM #229600). In two patients with SLD associated with hyperferritinemia, we established a clinical diagnosis of *HFE*‐related hemochromatosis. In two patients with associated dyslipidemia, a diagnosis of heterozygous familial hypobetalipoproteinemia due to *APOB* variants was defined, while in another one, a diagnosis of familial hypercholesterolemia due to *LDLR* variant was reached (Table 2).

In patient #1, a genetic diagnosis of hereditary fructose intolerance was established due to the presence of a homozygous pathogenic variant (NM_000035: p.A150P) in *ALDOB* gene. He was a 59‐year‐old male presenting with SLD and advanced fibrosis (LSM 28 kPa), increased liver enzyme levels (ASL 65 IU/L, ALT 126 IU/L, GGT 167 IU/L) and hyperferritinemia (600 ng/mL) with normal transferrin saturation. Hereditary fructose intolerance is an autosomal recessive disease characterised by liver and kidney failure following fructose ingestion, whose treatment consists of fructose‐free diet [25].

In patient #2, genetic analysis allowed to reach a diagnosis of CESD, an autosomal rare disease whose clinical manifestations include hepatic steatosis (which may evolve into hepatic fibrosis and micronodular cirrhosis), splenomegaly, mixed hyperlipidemia, hypoalphalipoproteinemia and premature atherosclerosis. This patient, a 37‐year‐old male with hyperferritinemia (318 ng/mL), hyperlipidemia (LDL 164 mg/dL), and hepatic steatosis, harboured a homozygous pathogenic splicing variant (NM_001127605.3: c.894G>A, p.Ser275_Gln298del) in *LIPA* gene [26, 27]. Moreover, he was compound heterozygous for p.C282Y/p.H63D variants in *HFE* gene, consistent with his phenotype.

In patient #3, a 59‐year‐old female presenting with a BMI of 35.6 kg/m^2^, hypertriglyceridemia (serum triglycerides: 231 mg/dL), SLD and hepatic fibrosis, and a positive family history, we identified a rare heterozygous pathogenic variant (NM_000237: p.G181D) in *LPL* gene, accounting for a genetic diagnosis of familial combined hyperlipidemia, characterised by increased levels of serum cholesterol and triglycerides. This patient had two additional rare heterozygous variants (NM_000443: p.N510S and p.L73V) in the *ABCB4* gene, possibly contributing to SLD [28]. Moreover, the p.N510S variant in *ABCB4* was associated to an increased risk of cardiovascular diseases in the UKBB cohort, further underling the need of cardiovascular evaluation and personalised management of this patients.

Patient #4 harboured a pathogenic variant (NM_000214: p.I1028T) in *JAG1* gene and was diagnosed with Alagille syndrome, an autosomal dominant genetic disorder characterised by extreme clinical heterogeneity [29]. This patient was a 60‐year‐old male with cirrhosis, type 2 diabetes, dyslipidemia and a positive family history of cirrhosis.

In two other patients, we identified rare variants allowing a suggested diagnosis of telomeropathies. In patient #5, a 57‐year‐old male with a history of cirrhosis associated with SLD and portal hypertension, who subsequently developed HCC, we identified a rare variant (NM_001193376: p.H412Y) in *TERT* gene, allowing a suggested diagnosis of autosomal dominant *TERT*‐associated telomerase syndrome. Loss‐of‐function germline mutations in *TERT* predispose to a spectrum of familial liver diseases characterised by steatosis and possible evolution to cirrhosis and HCC [30, 31]. Patient #6, a 64‐year‐old male with decompensated cirrhosis (LSM 26.6 kPa), portal hypertension and diabetes, was identified to have a rare variant (NM_001283009: p.D1017E) in *RTEL1* gene and a suggested diagnosis of *RTEL1*‐associated telomerase syndrome was done. RTEL1 is involved in maintaining telomere length, and heterozygous loss‐of‐function *RTEL1* variants have been associated with myelodysplasia and liver disease in adulthood [32].

In two patients with SLD and hyperferritinemia, a diagnosis of *HFE*‐related hemochromatosis was reached, due to compound heterozygosity for p.C282Y/p.H63D variants in *HFE* gene in one patient (#7) and to p.C282Y homozygosity in the other one (#8), accounting for their phenotype characterised by high ferritin levels with high transferrin saturation (300 ng/mL and 67%, respectively, in the first patient and 2189 ng/mL and 86% in the second one). Interestingly, in patient #8, the presence of two additional heterozygous rare variants in *TFR2* and *HJV* genes (NM_003227: p.R752H and NM_213653: p.G69_R70insG, respectively) could contribute to exacerbate the clinical phenotype, although these variants are presently classified as likely benign [33].

One patient with SLD and increased LDL levels (213 mg/dL), a 49‐year‐old man (#9), with a positive family history for hypercholesterolemia, harboured a heterozygous pathogenic variant (NM_000527: p.T434M) in *LDLR* gene, accounting for a genetic diagnosis of heterozygous familial hypercholesterolemia [34, 35].

Finally, in two patients (#10 and #11), we defined a genetic diagnosis of heterozygous *APOB*‐related Familial Hypobetalipoproteinemia (FHBL) due to the presence of heterozygous pathogenic/likely pathogenic variants in *APOB* gene (NM_000384.3: p.S2429X and c.3696 + 2 T>G, respectively). Both patients, two young 20‐years and 29‐years old males, presented with SLD, associated with hepatic fibrosis (LSM 9.9 kPa) in the second one. Serum LDL levels were 15 and 24 mg/dL, and serum triglycerides levels were 38 and 63 mg/dL, respectively. The circulating APOB levels were low (0.17 g/L and 0.25 g/L, respectively; normal range: 0.75–1.5 g/L), consistent with heterozygous FHBL [36]. Both patients had a positive family history for cirrhosis. Interestingly, patient #10 had also a high SLD‐PRS score (0.73; *PNPLA3* rs738409 genotype: GG) suggestive of increased genetic predisposition to SLD, possibly exacerbating the clinical phenotype.

The clinical features of patients with genetic variants possibly contributing to clinical phenotype are reported in the Data S1: Results section.

### 3.4 SLD‐PRS Determination

In the overall cohort, increased SLD‐PRS values (> 0.495) were detected in 17 patients (35%), 14 of whom (82%) had isolated SLD (Table 2 and Table S3). In 14/17 cases (82%), patients with a high SLD‐PRS also had clinically relevant variants, defining a genetic diagnosis in 5/14 (36%) or possibly contributing to the clinical phenotype in 9/14 (64%); three patients (18%) had only a high PRS score with no relevant variants (Figure 2A). The clinical features of patients with a high SLD‐PRS are reported in the Data S1: Results section.

### 3.5 Diagnostic Uptake, Predictors and Clinical Implications

Diagnostic uptake for Mendelian disorders did not differ according to the age, sex, family history or SLD‐PRS values (Table 3, left panel). On the other hand, SLD‐PRS values were associated with a negative family history (*p* = 0.04) and remained nearly associated after correction for sex and age at multivariable analysis (OR 0.6; 95% CI: 0.3–1.1; *p* = 0.07; Table 3, right panel).

**TABLE 3:** Independent determinants of diagnostic uptake and SLD‐PRS value in the overall cohort.

	Diagnostic uptake						High SLD‐PRS					
	Unadjusted			Multivariable model			Unadjusted			Multivariable model		
	OR	95% CI	*p*	OR	95% CI	*p*	OR	95% CI	*p*	OR	95% CI	*p*
Age, years	0.97	0.92–1	0.19	0.99	0.93–1.05	0.76	1	0.97–1	0.5	1	0.95–1.05	1
Sex, male	4.61	0.53–40.28	0.17	1.73	0.62–4.79	0.22	0.5	0.14–1.89	0.3	0.86	0.4–1.8	0.7
Family history, positive	1.67	0.49–4.27	0.19				0.29	0.08–0.98	0.04	0.6	0.3–1.1	0.07
Clinical phenotype			0.28			0.61			0.05			
SLD	0.65	0.26–1.63	0.40	0.82	0.28–2.39	0.79	2.11	0.83–5.41	0.06			
SLD & dyslipidemia	2.54	0.76–8.49	0.11	1.85	0.49–7	0.32	1.42	0.39–5.11	0.61			
High SLD‐PRS	1.8	0.46–7.1	0.39	1.39	0.66–2.92	0.35						

*Note:* Logistic regression analysis unadjusted and adjusted for the covariates reported in the models.

Abbreviations: SLD, steatotic liver disease; SLD‐PRS, polygenic risk score for steatotic liver disease.

Considering the presence of a high SLD‐PRS that captures the genetic predisposition to SLD due to carriage of common variants, alone or with rare VUS possibly contributing to clinical phenotype, the diagnostic uptake increased in the overall cohort (12 cases: +24%, *p* = 0.03), and in patients with isolated SLD (10 cases: +32%, *p* = 0.05) (Figure 2B).

The benefits of the genetic diagnoses established in the present study and their implications for clinical management are reported in Table 4.

**TABLE 4:** Impact of newly established genetic diagnoses on clinical management of patients.

Clinical diagnosis	Gene involved	Changes to clinical management
*HFE*‐related Hemochromatosis	*HFE*	Phlebotomy, study of iron overload target organs, hepatological follow up
Familial, Hypercholesterolemia, type 1	*LDLR*	Start lipid lowering therapy, evaluate cardiovascular risk factors, cardiologist referral
Cholesteryl ester storage disease (CESD)	*LIPA*	Potentiate dietary interventions, lipid lowering therapy, evaluate cardiovascular risk factors, evaluate enzyme replacement therapy, hepatological follow up
Combined hyperlipidemia	*LPL*	Lipid‐lowering therapy, evaluate cardiovascular risk factors
Heterozygous Familial Hypobetalipoproteinemia	*APOB*	Potentiate dietary interventions, vitamin E supplementation, hepatological follow up
*TERT*‐associated telomerase syndrome	*TERT*	Evaluate telomeropathies target organs other than the liver (lung function, signs of bone marrow involvement), hepatological follow up
*RTEL1*‐associated telomerase syndrome	*RTEL1*	Evaluate telomeropathies target organs other than the liver (lung function, signs of bone marrow involvement), hepatological follow up
Alagille syndrome	*JAG1*	Set specific treatment (ileal bile acid transporter inhibitors), hepatological follow up
Hereditary Fructose Intolerance	*ALDOB*	Set proper fructose‐free diet, hepatological follow up
Polygenic predisposition to SLD	*PNPLA3, TM6SF2, MBOAT7, GCKR, HSD17B13*	Enhance body weight interventions, HCC surveillance

Abbreviations: HCC, hepatocarcinoma; SLD, steatotic liver disease.

## 4 Discussion

In this study, we examined the diagnostic uptake and explored the clinical utility of NGS by a TS panel [19] in the diagnosis and management of adult patients with SLD, with or without systemic involvement, unexplained despite clinical, instrumental and histological work‐up by expert hepatologists. The main finding was that a genetic diagnosis of a Mendelian disorder, definite or suggested, was established in 22% of patients. In addition, the concomitant evaluation of genetic predisposition to SLD due to common variation, as captured by PRS‐SLD, increased the diagnostic uptake (+24%), reaching an overall diagnostic uptake of 47%.

The diagnostic yield of this study was comparable to those reported in smaller cohorts of adult patients with similar phenotypes of unexplained liver diseases, evaluated through WES [11, 12, 13, 14, 15]. A genetic diagnosis of a Mendelian disorder usually presenting in the paediatric age and therefore poorly investigated in adulthood was established in three cases. These findings lend further support to the role of NGS not only in confirming clinical diagnoses in patients with well‐defined phenotypes suggestive of genetic disorders but also in establishing a diagnosis in patients with subtle or atypical phenotypes. Results also contribute to understand and expand the spectrum of monogenic liver diseases with clinical presentation in adulthood, that is still currently underappreciated [10, 17].

The established genetic diagnoses had an impact on clinical management. They suggested a personalised follow‐up, as in patients with variants in *TERT* or *RTEL1* genes, who are more prone to develop HCC or extra‐hepatic organ failure (lung or bone marrow), [30, 31] targeted therapy, as for iron in patients with hemochromatosis and replacement therapy for the one with CESD, or additional specialist referrals. Additionally, benefits of a genetic diagnosis also included the ability to guide familial testing and obtain an early diagnosis of affected family members [7, 15, 37].

In 59% of patients, although a genetic diagnosis was not reached, rare variants of unknown significance, but with a high likelihood of disrupting protein function, were identified in genes involved in disease pathogenesis, contributing to getting insight into liver damage pathophysiology in individual cases. For instance, this was the case of one rare heterozygous VUS in *ABHD5* gene that could contribute to explain SLD, as heterozygosity for loss‐of‐function mutations hampering lipid droplets hydrolysis has been proposed to drive genetics steatosis [38]. Larger studies are needed to further support the role of heterozygous carriage of rare variants in disease predisposition. For those variants identified here which were sufficiently represented in population‐based UKBB cohort, we found an association with liver biomarkers in two of them in *ABCB4* and *APOB* genes, strongly supporting their role in phenotype determination.

Interestingly, in one patient with a genetic diagnosis of *HFE*‐related hemochromatosis but with an atypical phenotype, additional low‐frequency variants in other hemochromatosis genes (*TFR2* and *HJV*) were detected, contributing to the clinical phenotype, underlying the additional potential role of genetic heterogeneity detected by NGS to explain the penetrance and phenotypic expression of classical genetic diseases [39].

A novelty of the present study was the combined evaluation of the contribution of common variants, as captured by SLD‐PRS, which was remarkably associated with a negative family history. This finding is in line with literature being family history of severe disease in first‐degree relatives and polygenic risk independent measures, providing complementary information on inherited disease susceptibility. Family history captures both genetic (mainly carriage of rare genetic variants with a high penetrance) and non‐genetic factors, and may therefore be suggestive of inherited Mendelian disorders. PRS captures each person's unique combinations of common, disease‐associated variants, including genetic variations not shared by single relatives [40]. A large fraction of patients with SLD had a high SLD‐PRS value (> 0.495, that is over 95th percentile of general population), with or without the concomitant presence of rare variants. The combined evaluation of rare and common variation, as captured by PRS, may therefore increase the diagnostic uptake for patients with SLD identifying a subset of individuals at high genetic risk, who could benefit from a personalised surveillance program, as it has been demonstrated for other conditions, including cardiovascular disease and breast cancer [41]. Specifically, as SLD‐PRS has been associated with an increased risk for HCC independently of cirrhosis, enhanced surveillance could be offered also in patients with clinical risk factors and a high genetic risk even in the absence of cirrhosis [20]. Moreover, SLD‐PRS determination could identify individuals at high risk before they show clinical signs or symptoms, such as in asymptomatic relatives of individuals with clinically overt SLD [7, 42]. For these at‐risk individuals, a genetic counselling performed in a multidisciplinary team, including hepatologists and clinical geneticists, could be offered to set up a specific follow‐up.

Strengths of the present study include the larger samples size than previous ones, [11, 16] the first large application of TS strategy allowing higher coverage and UTR regions evaluation vs. the WES approach, and assessment of the complementary role of PRS calculation to detect genetic predisposition. Moreover, the selection of a homogeneous cohort of adult patients with SLD with suspected genetic contribution, excluding those with SLD due to other etiologies, such as alcohol, viral infections or drugs, further allowed to evaluate the impact of genetic variants on disease pathogenesis. These findings should then be extended to other populations, including the paediatric one, to improve the clinical diagnosis and management.

Limitations include the still relatively small sample size that limit the statistical power of the study and the generalizability of the results; moreover, the clinical follow‐up we performed is limited; a long‐term follow‐up would provide a more comprehensive understanding of the clinical benefits of genetic diagnosis, for the patients and their first‐degree relatives too. Finally, a technical limit is the lack of possibility to examine the contribution of copy number variants (CNV), which could have led to a slight underestimation of the overall diagnostic uptake achievable with the most recent approaches.

All in all, using a customised TS specific for liver diseases in a cohort of adult patients with SLD, we obtained a diagnostic uptake for Mendelian disorders of 22%, overlapping with that reported for WES in cohorts of adult patients with similar phenotypes. The complementary evaluation of common variants predisposing to SLD, as captured by SLD‐PRS, could increase the diagnostic uptake to 47%.

In conclusion, although additional validation in larger multiethnic cohorts are still necessary to strengthen these results, in line with previous findings [12, 15, 16] and in keeping with clinical guidelines [4, 43], these data support the use of NGS‐TS analysis, with complementary evaluation of rare variants and PRS stratification in the diagnostic work‐up of adult patients with SLD, especially in those with a family history of severe disease, or absence of metabolic triggers, early presentation or a severe and systemic phenotype.

## Author Contributions

L.R. conceived and designed the study, analysed and interpreted NGS data, drafted the initial manuscript and revised the initial and final manuscript; S.P. recruited patients and drafted the initial manuscript; V.M. performed NGS and Sanger analysis, drafted the initial manuscript; J.R. performed NGS and Sanger analysis; F.M., H.E.T., O.J. and M.R.D.F. performed bioinformatic analysis; C.B. and G.P. recruited patients; S.R. revised the final manuscript; D.P. overviewed the study and revised the final manuscript; L.V. conceived and designed the study, overviewed and funded the study and revised the initial and final manuscript. All authors contributed to the manuscript and approved the submitted version.

## Ethics Statement

The study protocol was approved by the Ethical Committee of Fondazione IRCCS Ca′ Granda Ospedale Maggiore Policlinico, Milan (CE 125_2018bis). Written informed consent for genetic analysis was obtained from each patient.

## Conflicts of Interest

The authors declare no conflicts of interest. L.V. has received speaking fees from: Viatris, Novo Nordisk, G.S.K., consulting fees from: Novo Nordisk, Pfizer, Boehringer Ingelheim, Resalis and unrestricted grant support from: Gilead.

## Supporting information


Figure S1.

Data S1.

Table S1.

Table S2.

Table S3.

Table S4.

## Data Availability

The datasets used and/or analysed during the current study are available from the corresponding author on reasonable request.

## References

[1] M. E. Rinella , J. V. Lazarus , V. Ratziu , et al., “A Multisociety Delphi Consensus Statement on New Fatty Liver Disease Nomenclature,” Journal of Hepatology 79, no. 6 (2023): 1542–1556. 37364790 10.1016/j.jhep.2023.06.003

[2] M. Israelsen , S. Francque , E. A. Tsochatzis , and A. Krag , “Steatotic Liver Disease,” Lancet 2 (2024): 1761–1778. 10.1016/S0140-6736(24)01811-7 39488409

[3] P. Ginès , L. Castera , F. Lammert , et al., “Population Screening for Liver Fibrosis: Toward Early Diagnosis and Intervention for Chronic Liver Diseases,” Hepatology 75, no. 1 (2022): 219–228. 34537988 10.1002/hep.32163

[4] European Association for the Study of the Liver (EASL); European Association for the Study of Diabetes (EASD); European Association for the Study of Obesity (EASO); European Association for the Study of the Liver (EASL) , “EASL‐EASD‐EASO Clinical Practice Guidelines on the Management of Metabolic Dysfunction‐Associated Steatotic Liver Disease (MASLD),” Journal of Hepatology 81, no. 3 (2024): 492–542. 38851997 10.1016/j.jhep.2024.04.031

[5] L. Henry , J. Paik , and Z. M. Younossi , “Review Article: The Epidemiologic Burden of Non‐Alcoholic Fatty Liver Disease Across the World,” Alimentary Pharmacology & Therapeutics 56, no. 6 (2022): 942–956. 35880713 10.1111/apt.17158

[6] P. Golabi , J. Paik , N. Fukui , C. T. Locklear , L. de Avilla , and Z. M. Younossi , “Patients With Lean Nonalcoholic Fatty Liver Disease Are Metabolically Abnormal and Have a Higher Risk for Mortality,” Clinical Diabetes 37, no. 1 (2019): 65–72. 30705499 10.2337/cd18-0026 PMC6336127

[7] S. Pelusi , L. Ronzoni , J. Rondena , et al., “Prevalence and Determinants of Liver Disease in Relatives of Italian Patients With Advanced MASLD,” Clinical Gastroenterology and Hepatology 22, no. 11 (2024): 2231–2239.e4. 38216023 10.1016/j.cgh.2023.12.033

[8] E. Gao , J. Hercun , T. Heller , and S. Vilarinho , “Undiagnosed Liver Diseases,” Translation Gastroenterology and Hepatology 6 (2021): 28. 10.21037/tgh.2020.04.04 PMC7829073 33824932

[9] L. Valenti and L. Ronzoni , “Genetics: A New Clinical Tool for the Hepatologist,” Liver International 42, no. 4 (2022): 724–726. 35289075 10.1111/liv.15205

[10] D. H. Chung , M. Zheng , A. E. Bale , and S. Vilarinho , “Hepatology Genome Rounds: An Interdisciplinary Approach to Integrate Genomic Data Into Clinical Practice,” Journal of Hepatology 79, no. 4 (2023): 1065–1071. 37011712 10.1016/j.jhep.2023.03.030 PMC10523901

[11] A. Hakim , X. Zhang , A. DeLisle , et al., “Clinical Utility of Genomic Analysis in Adults With Idiopathic Liver Disease,” Journal of Hepatology 70, no. 6 (2019): 1214–1221. 31000363 10.1016/j.jhep.2019.01.036 PMC6526061

[12] X. F. Kong , K. Bogyo , S. Kapoor , et al., “The Diagnostic Yield of Exome Sequencing in Liver Diseases From a Curated Gene Panel,” Scientific Reports 13, no. 1 (2023): 21540. 38057357 10.1038/s41598-023-42202-1 PMC10700603

[13] J. S. Nayagam , P. Foskett , S. Strautnieks , et al., “Clinical Phenotype of Adult‐Onset Liver Disease in Patients With Variants in ABCB4, ABCB11, and ATP8B1,” Hepatology Communication 6, no. 10 (2022): 2654–2664. 10.1002/hep4.2051 PMC9512461 35894240

[14] S. Vilarinho , V. Ajmera , M. Zheng , and R. Loomba , “Emerging Role of Genomic Analysis in Clinical Evaluation of Lean Individuals With NAFLD,” Hepatology 74, no. 4 (2021): 2241–2250. 34233030 10.1002/hep.32047 PMC8463418

[15] S. Pelusi , L. Ronzoni , F. Malvestiti , et al., “Clinical Exome Sequencing for Diagnosing Severe Cryptogenic Liver Disease in Adults: A Case Series,” Liver International 42, no. 4 (2022): 864–870. 35132767 10.1111/liv.15185

[16] M. Zheng , A. Hakim , C. Konkwo , et al., “Advancing Diagnosis and Management of Liver Disease in Adults Through Exome Sequencing,” eBioMedicine 95 (2023): 104747. 37566928 10.1016/j.ebiom.2023.104747 PMC10433007

[17] L. Valenti , S. Pelusi , and G. Baselli , “Whole Exome Sequencing for Personalized Hepatology: Expanding Applications in Adults and Challenges,” Journal of Hepatology 71, no. 4 (2019): 849–850. 31362836 10.1016/j.jhep.2019.06.008

[18] M. Zheng , G. Allington , and S. Vilarinho , “Genomic Medicine for Liver Disease,” Hepatology 76, no. 3 (2022): 860–868. 35076957 10.1002/hep.32364 PMC10460497

[19] L. Ronzoni , I. Marini , G. Passignani , et al., “Validation of a Targeted Gene Panel Sequencing for the Diagnosis of Hereditary Chronic Liver Diseases,” Frontiers in Genetics 14 (2023): 1137016. 37388930 10.3389/fgene.2023.1137016 PMC10300275

[20] C. Bianco , O. Jamialahmadi , S. Pelusi , et al., “Non‐Invasive Stratification of Hepatocellular Carcinoma Risk in Non‐Alcoholic Fatty Liver Using Polygenic Risk Scores,” Journal of Hepatology 74, no. 4 (2021): 775–782. 33248170 10.1016/j.jhep.2020.11.024 PMC7987554

[21] L. Valenti , E. Corradini , L. A. Adams , et al., “Consensus Statement on the Definition and Classification of Metabolic Hyperferritinaemia,” Nature Reviews. Endocrinology 19, no. 5 (2023): 299–310. 10.1038/s41574-023-00807-6 PMC9936492 36805052

[22] S. Richards , N. Aziz , S. Bale , et al., “Standards and Guidelines for the Interpretation of Sequence Variants: A Joint Consensus Recommendation of the American College of Medical Genetics and Genomics and the Association for Molecular Pathology,” Genetics in Medicine 17, no. 5 (2015): 405–424. 25741868 10.1038/gim.2015.30 PMC4544753

[23] G. A. Baselli , O. Jamialahmadi , S. Pelusi , et al., “EPIDEMIC Study Investigators. Rare *ATG7* Genetic Variants Predispose Patients to Severe Fatty Liver Disease,” Journal of Hepatology 77, no. 3 (2022): 596–606. 35405176 10.1016/j.jhep.2022.03.031

[24] L. Balcar , B. Scheiner , M. Urheu , et al., “Alpha‐1 Antitrypsin Pi*Z Allele Is an Independent Risk Factor for Liver Transplantation and Death in Patients With Advanced Chronic Liver Disease,” JHEP Report 4, no. 11 (2022): 100562. 10.1016/j.jhepr.2022.100562 PMC9513767 36176936

[25] F. C. Pinheiro , F. Sperb‐Ludwig , and I. V. D. Schwartz , “Epidemiological Aspects of Hereditary Fructose Intolerance: A Database Study,” Human Mutation 42, no. 12 (2021): 1548–1566. 34524712 10.1002/humu.24282

[26] L. Arnaboldi , A. Ossoli , E. Giorgio , et al., “LIPA Gene Mutations Affect the Composition of Lipoproteins: Enrichment in ACAT‐Derived Cholesteryl Esters,” Atherosclerosis 297 (2020): 8–15. 32058863 10.1016/j.atherosclerosis.2020.01.026

[27] L. Pisciotta , G. Tozzi , L. Travaglini , et al., “Molecular and Clinical Characterization of a Series of Patients With Childhood‐Onset Lysosomal Acid Lipase Deficiency. Retrospective Investigations, Follow‐Up and Detection of Two Novel LIPA Pathogenic Variants,” Atherosclerosis 265 (2017): 124–132. 28881270 10.1016/j.atherosclerosis.2017.08.021

[28] J. S. Dron and R. A. Hegele , “Genetics of Hypertriglyceridemia,” Frontiers in Endocrinology 11 (2020): 455. 32793115 10.3389/fendo.2020.00455 PMC7393009

[29] T. J. Kohut , M. A. Gilbert , and K. M. Loomes , “Alagille Syndrome: A Focused Review on Clinical Features, Genetics, and Treatment,” Seminars in Liver Disease 41, no. 4 (2021): 525–537. 34215014 10.1055/s-0041-1730951

[30] B. Donati , A. Pietrelli , P. Pingitore , et al., “Telomerase Reverse Transcriptase Germline Mutations and Hepatocellular Carcinoma in Patients With Nonalcoholic Fatty Liver Disease,” Cancer Medicine 6, no. 8 (2017): 1930–1940. 28677271 10.1002/cam4.1078 PMC5548883

[31] B. Donati and L. Valenti , “Telomeres, NAFLD and Chronic Liver Disease,” International Journal of Molecular Sciences 17, no. 3 (2016): 383. 26999107 10.3390/ijms17030383 PMC4813240

[32] S. R. Cardoso , A. C. M. Ellison , A. J. Walne , et al., “Myelodysplasia and Liver Disease Extend the Spectrum of RTEL1 Related Telomeropathies,” Haematologica 102, no. 8 (2017): e293–e296. 28495916 10.3324/haematol.2017.167056 PMC6643735

[33] A. Piperno , S. Pelucchi , and R. Mariani , “Inherited Iron Overload Disorders,” Translation Gastroenterology and Hepatology 5, no. 5 (2020): 25. 10.21037/tgh.2019.11.15 PMC7063521 32258529

[34] H. E. Ison , S. L. Clarke , and J. W. Knowles , “Familial Hypercholesterolemia,” GeneReviews 20 (2014): 1993–2023.

[35] A. C. Sturm , J. W. Knowles , S. S. Gidding , et al., “Clinical Genetic Testing for Familial Hypercholesterolemia: JACC Scientific Expert Panel,” Journal of the American College of Cardiology 72, no. 6 (2018): 662–680. 30071997 10.1016/j.jacc.2018.05.044

[36] J. R. Burnett , A. J. Hooper , and R. A. Hegele , “APOB‐Related Familial Hypobetalipoproteinemia,” GeneReviews 20 (2021): 1993–2023. 33983694

[37] N. Tamaki , N. Ahlholm , P. K. Luukkonen , et al., “Risk of Advanced Fibrosis in First‐Degree Relatives of Patients With Nonalcoholic Fatty Liver Disease,” Journal of Clinical Investigation 132, no. 21 (2022): e162513. 36317632 10.1172/JCI162513 PMC9621132

[38] L. Youssefian , H. Vahidnezhad , A. H. Saeidian , et al., “Inherited Non‐Alcoholic Fatty Liver Disease and Dyslipidemia due to Monoallelic ABHD5 Mutations,” Journal of Hepatology 71, no. 2 (2019): 366–370. 30954460 10.1016/j.jhep.2019.03.026 PMC7285838

[39] L. Valenti , S. Pelusi , and L. Ronzoni , “Hereditary Hemochromatosis,” in Comprehensive Guide to Hepatitis Advances, eds. W.‐K. Seto and M. Eslam (London, UK: Elsevier, 2023), 443–458.

[40] N. Mars , J. V. Lindbohm , P. Della Briotta Parolo , et al., “Systematic Comparison of Family History and Polygenic Risk Across 24 Common Diseases,” American Journal of Human Genetics 109, no. 12 (2022): 2152–2162. 36347255 10.1016/j.ajhg.2022.10.009 PMC9748261

[41] A. V. Khera , M. Chaffin , K. G. Aragam , et al., “Genome‐Wide Polygenic Scores for Common Diseases Identify Individuals With Risk Equivalent to Monogenic Mutations,” Nature Genetics 50, no. 9 (2018): 1219–1224. 30104762 10.1038/s41588-018-0183-z PMC6128408

[42] C. Bianco , F. Tavaglione , S. Romeo , and L. Valenti , “Genetic Risk Scores and Personalization of Care in Fatty Liver Disease,” Current Opinion in Pharmacology 61 (2021): 6–11. 34537584 10.1016/j.coph.2021.08.014

[43] J. S. Nayagam , S. Masson , J. W. Ferguson , W. Griffiths , and D. Joshi , “Where Does Genetic Testing Fit in the Diagnostic Pathway of Patients With Cryptogenic Cirrhosis?,” Journal of Hepatology 79, no. 5 (2023): e211–e213. 37380107 10.1016/j.jhep.2023.05.049

