# Supplementary material for: Diagnostic Uptake of Targeted Sequencing in Adults With Steatotic Liver Disease and a Suspected Genetic Contribution
Source: Liver Int. 2025 Feb 13;45(3):e70010. doi: 10.1111/liv.70010 (PMC11822878; doi:10.1111/liv.70010)
Supplement: Supplementary file 1 — Figure S1. [file LIV-45-0-s001.pdf]

First and Last name:

Date of birth: **Age:** **Sex:**

Clinical indication for genetic analysis:

Biological sample: DNA extracted from peripheral blood

Genetic analysis:

-Next Generation Sequencing (NGS) of 82 liver and lipid metabolism-related genes. Technical features and the list of the analyzed genes are reported at the bottom of the medical report.

-Polygenic risk score for steatotic liver disease (SLD-PRS) determination, based on *PNPLA3* rs738409 C>G, *TM6SF2* rs58542926 C>T, *MBOAT7* rs641738 C>T, *GCKR* rs1260326 C>T and *HSD17B13* rs72613567 T>TA

Results:

Genetic variants identified:

Gene	Variant (HGVS)	Zigosity	Frequency (ExAC)	ACMG classification	Associated disease, inheritance
XXX	NM_<br>c.(….); p.(….)	Heterozygous	0.0002	Pathogenic	XXXX, AR/AD

Genotype *PNPLA3* rs738409:
TM6SF2 rs58542926:
MBOAT7 rs641738:
GCKR rs1260326:
HSD17B13 rs72613567:

SLD-PRS: …. (VN≤0.495)

Mean coverage: …X; bases with a coverage >20X: 98% For the analysis only the regions with a coverage >20X have been considered

Data interpretation and conclusion:

……

NGS technical data: NGS was performed using a customized Haloplex target panel kit (Agilent) on MiSeq (Illumina) platform. The coding regions (± 25bp) of 82 genes were analyzed; for specific genes, available under request, the UTR regions were also included. Bioinformatic analysis was performed using the SureCall software (Agilent) and the sequences were aligned to the GRCh37 reference genome. The variants were selected and annotated using wAnnovar tool. Allele frequency was defined according to gnomAD (Genome Aggregation Database). Only genetic variants with appropriate quality and depth were considered.
Technical limits of NGS analysis: This NGS technology does not allow to identify single exon, multiexonic or whole gene deletions and duplications, complex rearrangements and mutations due to the expansion of repeated regions. The presence of pseudogenes could reduce the sensitivity and specificity of the test

Notes: Variants interpretation is based on current scientific knowledges and the use of database like ClinVar, Human Gene Mutation Database, Varsome and specific disease databases. It is possible that a variant could not be recognized as causative of clinical disease due to lack or incomplete scientific knowledges. Variant's nomenclature and classification could vary according to new scientific evidences. The non-identification of pathogenic variants doesn't exclude a possible genetic cause; it is possible that some variants are currently not known or that are located in genes not evaluated with this sequencing analysis.

Analyzed genes: *ABCA1, ABCB11, ABCB4, ABHD5, AGL, AGPAT2, ALDOB, ALMS1, APOA1, APOA5, APOB, APOC2, APOE, ASL, ATG7, ATP7B, ATP8B1, BMP6, BSCL2, CAV1, CAVIN1, CELA2A, CETP, CIDEC, CP, DGUOK, FAH, FTL, G6PC, GAA, GBA, GBE, GCKR, GPD1, GPIHBP1, HAMP, HFE, HJV, HSD17B13, IRF3, JAG1, LCAT, LDLR, LDLRAP1, LIPA, LIPC, LIPE, LMF1, LMNA, LPL, MAN2B1, MBOAT7, MTTP, NMBR, NPC1, NPC2, NR1H4, PCSK7, PCSK9, PHKA2, PHKB, PHKG2, PLIN1, PNPLA2, PNPLA3, PPARG, PYGL, RTEL1, SCARB1, SERPINA1, SLC11A2, SLC25A13, SLC2A2, SLC37A4, SLC40A1, SMPD1, TERC, TERT, TF, TFR2, TJP2, TM6SF2*
