# Supplementary material for: Diagnostic Uptake of Targeted Sequencing in Adults With Steatotic Liver Disease and a Suspected Genetic Contribution
Source: Liver Int. 2025 Feb 13;45(3):e70010. doi: 10.1111/liv.70010 (PMC11822878; doi:10.1111/liv.70010)
Supplement: Supplementary file 2 — Data S1. [file LIV-45-0-s006.docx]

**Diagnostic uptake of targeted sequencing in adults with steatotic liver disease and a suspected genetic contribution**

Luisa Ronzoni^1^, Serena Pelusi^1^, Vittoria Moretti^1^, Francesco Malvestiti^2^, Hadi Eidgah Torghabehei^3^, Oveis Jamialahmadi^4^, Jessica Rondena^1^, Giulia Periti^1^, Cristiana Bianco^1^, Maria Rosaria De Filippo^3^, Stefano Romeo^4,5,6^, Daniele Prati^1^, Luca Valenti^1,2,3^

**Table of contents**

Supplementary methods ………………………………………………………………………….2

Supplementary results ……………………………………………………………………………5

Supplementary references ……………………………………………………………………….10

Supplementary tables ……………………………………………………………………………13

Supplementary figure ………………………………………………….………………………...14

Supplementary file …………………………………………………….………………………...14

**SUPPLEMENTARY METHODS**

**Study cohort**

The following data were collected for each patient: a) demographic data, including sex, age at the time of enrollment, family history for liver disease and ethnicity; b) clinical data, including BMI, the presence of type 2 diabetes (T2D) and of liver steatosis, fibrosis or cirrhosis, evaluated through abdominal ultrasound (US), vibration controlled transient elastography (VCTE) by Fibroscan or liver biopsy, as appropriated. Liver stiffness measurement (LSM) by VCTE has been reported as a parameter of possible advanced liver fibrosis or cirrhosis (≥8 kPa or ≥12.5 kPa respectively); c) biochemical liver tests (AST, ALT, GGT) and metabolic parameters (LDL plasma levels, triglycerides, ferritin and transferrin saturation). Two cases detailed in Supplementary results section have been already included in a previous publication describing a heterogeneous cohort of adult patients with cryptogenic liver disease ^1^ and were analyzed by both TS and WES approach, in this study and in the previous one, respectively.

**NGS Targeted Sequencing**

DNA was extracted from peripheral blood and quantified by a Qubit 2.0 analyzer using the Qubit dsDNA BR Assay Kit (Thermo-Fisher, Waltham, MA, USA). Sample purity was evaluated using a Nanodrop 1000 spectrophotometer (Thermo-Fisher, Waltham, MA, USA) and integrity was assessed by gel electrophoresis.

Amplicon libraries were prepared from genomic DNA using the HaloPlex Target Enrichment System specific for the designed targeted panel (Agilent, Cernusco sul Naviglio, Milan, Italy), according to the manufacture's protocol. Sequencing was performed on MiSeq platform (Illumina, San Diego, CA). FASTQ files were analyzed using SureCall version 4.2.2 (Agilent, Cernusco sul Naviglio, Milan, Italy). Briefly, adapter sequences and lower-quality bases were removed; reads were aligned to reference genome (GRCh37-hg19) using Burrows Wheeler Aligner (BWA)-MEM algorithm. A coverage depth cutoff of 20x in more than 98% of the targeted region was applied. Variant calling was performed using the algorithm SNPPET SNP of SureCall. The obtained Variant Call Format (vcf) files were analyzed and annotated in wANNOVAR server ([http://wannovar.usc.edu](http://wannovar.usc.edu/)).

**Sanger Sequencing**

The specific PCR primers were designed using both Primer Design Tool-NCBI (<https://www.ncbi.nlm.nih.gov/tools/primer-blast/>) and Primer3 software (<https://primer3.ut.ee/>); primers sequences are available upon request. Amplicons were sequenced using the Big Terminator v3.1 cycle sequencing kit (Applied Biosystems™, Thermo-Fisher, Waltham, MA, USA) and a Sanger Sequencing 3500 Dx Series Genetic Analyzer (Applied Biosystems™, Thermo-Fisher, Waltham, MA, USA). Sequences were first analyzed using the Sequencing Analysis Software v7.0 (Applied Biosystems™, Thermo-Fisher, Waltham, MA, USA) and then compared to reference genome using BLAST software (https://blast.ncbi.nlm.nih.gov/Blast.cgi).

**Clinical report generation**

Utilizing R software version 4.3.1, an automated report generation protocol has been developed. Critical to this process is the employment of the 'tidyverse' package, version 2.0.0, which facilitates data manipulation and the presentation of variant data in tabular form within the report. Complementary to this, the 'officer' package, version 0.6.3, alongside 'shiny', version 1.7.5.1, are instrumental in the composition of text and graphical content, as well as in the production of documents in .docx format suitable for dissemination to medical professionals.

The report initiates with demographic and clinical data pertaining to the patient, including date of birth, age, and sex, subsequently progressing to a detailed account of the analytical methodology employed. It presents the outcome of the analysis in a structured table delineating gene names, variant identifiers, reference sequences, annotations based on the Human Genome Variation Society (HGVS) nomenclature, amino acid alterations, and the clinical implications of the identified variants. Moreover, the document delineates genes contributing to polygenic risk scores. A dedicated section is available for clinicians to impart their expert interpretation and remarks concerning the individual case under review (Figure S1).

**UK Biobank validation**

For variants validation, we used 500K release of WES covering 200,625 individuals who had undergone exome-sequencing with 20x sequence coverage on ~96% of sites and mapped to GRCh38 genome assembly using OQFE pipeline ^2^. The association between selected variants and UKBB traits was examined using linear regression for continuous traits or Firth’s penalized likelihood logistic regression (logistf R package) for binary traits, and adjusted for age, sex, and FDR.

**SUPPLEMENTARY RESULTS**

**Rare variants possibly contributing to clinical phenotype**

In 29 patients, rare variants possibly contributing to clinical phenotype were detected (Supplementary Table 3). Patient #12 was a 55-year-old female presenting with type 2 diabetes, cirrhosis (LSM 75 kPa), portal hypertension and recurrent episodes of hepatic encephalopathy and esophageal varices bleeding; subsequently she underwent liver transplantation. She was found to harbor a missense heterozygous variant (NM_016006: p.P77Q) in *ABHD5* gene, annotated as VUS according to ACMG classification, possibly contributing to *ABHD5*-related SLD, mainly characterized by the presence of steatohepatitis and fibrosis, diabetes and dyslipidemia ^3^. Of note, the patient had a high SLD-PRS value and was homozygous for *PNPLA3* rs738409 p.I148M; due to the likely interaction between *ABHD5* and *PNPLA3* ^4^, it could be speculated that both these variants contributed to the clinical phenotype.

In five patients, genetic analysis detected the presence of rare heterozygous variants in genes involved in bile acid transport and metabolism, namely *TJP2* (#13: NM_001170415: p.R24H), *ABCB4* (NM_000443.4: p.M676L and p.E1099G, #14 and #15 respectively), *ATP8B1* (#16: NM_005603: p.I393V), and *NR1H4* (#17: NM_001206993.2: p.A35V). Patients #15 and #16 have been already previously reported ^1^. Patient #13 was a 46-year-old woman with a positive history of intrahepatic cholestasis of pregnancy (ICP) in two consecutive pregnancies. At the age of 46, she presented with an acute cholestatic episode of jaundice and pruritus, with increased total bilirubin level (18.8 mg/dL) and aminotransferases (AST 469 IU/L, ALT 798 IU/L). In the previous 5 months she had undertaken a ketogenic diet, with a 35-kilogram drop in body weight. She was found to have a rare heterozygous VUS (NM_001170415: p.R24H) in *TJP2* gene, a homozygous variant in the *ABCB4* gene (NM_000443: p. N168N) and a heterozygous one in *ABCB11* gene (NM_003742: p.V444A). *TJP2* codes for the Tight Junction Protein 2, which is part of the membrane-associated guanylate cyclase family. Loss-of-function variants in *TJP2* lead to severe cholestatic liver disease; heterozygous predisposing variants have been detected in women with ICP ^5^. The variants in *ABCB4* and *ABCB11* genes have been previously reported as common variants predisposing to ICP and drug-induced liver injury (DILI) ^6^. The other four patients, three overweight (#14, #16, #17) and one lean male, presented with increased liver enzymes (ALT and GGT) and SLD, that was associated with hepatic fibrosis in two cases (#14 and #16 with LSM 9.9 and 8.7 kPa, respectively). *ABCB4* is a member of the ATP-binding cassette (ABC) transporter family and encodes for the protein MDR3, which plays a role in phospholipid transport from hepatocytes into bile. *ATP8B1* encodes for FIC1, a member of the P-type ATPases, ATP-dependent membrane transporters known as phospholipid “flippases” that translocate phosphatidylserine into hepatocyte from lumen. *NRH1H4* encodes for farnesoid X receptor (FXR), which regulates the expression of bile salt export pump (BSEP) ^7^. Biallelic variants in these genes are associated with cholestatic diseases (MDR3 deficiency, benign recurrent intrahepatic cholestasis, progressive familial intrahepatic cholestasis type 5). However, heterozygous variants in cholestasis-related genes have been described as contributors to the development of liver disease, at least in adult-onset disease ^8^; our data support this hypothesis.

Four patients, three lean subjects (#18, #19, and #20) and one overweight female (#21), harbored rare variants in *APOB* gene (NM_000384: p.T3047M, p.V730I, p.P2821L, and p.D1113H respectively). *APOB* variants, beside to be associated with altered lipid levels, have been associated to an increased risk of HCC development ^9^ and are an emerging cause of lean NASH/MASH ^10,11^; of interest, p.V730I variant was associated in UKBB with chronic liver diseases, further supporting its role in disease pathogenesis. Patient #20, presenting with increased LDL levels (247 mg/dL) and SLD, beside the rare heterozygous variant in *APOB* gene harbored another heterozygous variant in *LIPA* gene (NM_001127605.3: p.K2N). It could be speculated that, taken together, these variants could contribute to the clinical phenotype of hypercholesterolemia and increased liver enzymes in SLD. Of note, the patient had a high SLD-PRS score that could have contributed to the hepatic phenotype.

Heterozygous variants in *SERPINA1* gene have been identified in three patients. In one case (#22), the patient harbored the PiZ allele (NM_000295: p.E366K), known to be associated with increased risk of liver involvement ^12^, as also confirmed by UKBB validation; in another one (#23), we found the PiI allele (NM_000295: p.R63C) that, associated with a high SLD-PRS score, could contribute to clinical phenotype; in the last one (#24) genetic analysis detected a very rare missense variant (NM_000295: p.L65P), classified as likely pathogenic according to ACMG classification, whose role in phenotype determination could not be ruled out. Of note, the last patient also harbored a rare VUS in *RTEL1* gene (NM_001283009: p.D1261E) possibly contributing to clinical phenotype.

In six other patients with isolated SLD, heterozygous rare variants in genes involved in lipid metabolism (*PNPLA3*, *PCSK7* in combination with VUS in *APOB* and *ABCB11*, and *GCKR,*) or lysosomal storage diseases (*SMPD1* and *MAN2B1*) were identified, possibly contributing to explain the pathogenesis of liver disease.

In seven patients with SLD and hyperferritinemia, rare variants in genes involved in iron metabolism, alone or in combination to other variants in genes associated to liver diseases, were detected.

In four patients, heterozygous variants in *HFE* gene were associated with heterozygous variants in other genes. Patient #31, heterozygous for the p.C282Y variant in *HFE* gene, also carried a heterozygous polymorphism in *TF* gene (NM_001063: p.G277S). Variants in this gene, encoding for serum transferrin, have pleiotropic effects on serum ferritin or transferrin saturation ^13,14^; the combined presence of these variants could explain the patient phenotype (ferritin levels: 928 ng/mL, transferrin saturation 37%). Three patients were heterozygous for p.H63D variant. In patient #32, a 66-year-old female with hyperferritinemia (ferritin levels: 919 ng/mL; transferrin saturation 44%) and liver fibrosis (stiffness 39kPa), a likely pathogenic variant in *CP* gene (NM_000096.4: p.P477L) was detected. *CP* variants have been associated with hyperferritinemia, hepatic siderosis, and more severe liver fibrosis in patients with SLD, presumably accounting for this patient phenotype ^15^. In patient #33 the *HFE* p.H63D variant was associated with a heterozygous VUS in *ABCB4* gene and in patient #34 with heterozygous VUS in *SERPINA1* (NM_000295: p.A66V) and *ATP7B* (NM_000053: p.I116T) genes, respectively associated with alpha-1 Antitrypsin deficiency and Wilson disease. It could be hypothesized that the combined presence of these variants contributed to the clinical phenotype, mainly characterized by high ferritin levels with normal transferrin saturation and liver steatosis ^16^.

Patients #35 and #36 had heterozygous variants in *PCSK7* and *NMBR* genes. An enrichment in rare and low frequency loss-of-function *NMBR* variants has been demonstrated in patients with unexplained iron overload, especially when associated with SLD, and the NMBR receptor has been shown to be involved in the upregulation of hepcidin release from hepatocytes in response to saturated transferrin ^17^. The combined presence of variants in *PCSK7* and *NMBR* genes could contribute to the phenotype. Moreover, p.R310W in *NMBR* (NM_002513) has been associated to severe liver disease trait in UKBB, further supporting its role in phenotype determination. Finally, one patient (#37) had a heterozygous VUS in *DGOUK* gene, associated with Mitochondrial DNA Depletion syndrome, characterized by liver failure and hemochromatosis ^18^, possibly contributing to clinical phenotype.

Finally, in three patients with SLD and dyslipidemia we identified rare variants in gene involved in lipid metabolism, possibly contributing to understand the disease pathogenesis although the genetic data was not sufficient to reach a diagnosis.

Patient #38, a 30-year-old male with low LDL and triglycerides levels (45 and 36 mg/dL, respectively), had a nonsense variant (NM_000041: p.E98X) in *APOE* gene, not previously reported in literature. ApoE is a key regulator of plasma lipid levels: it modulates the receptor-mediated clearance, lipolytic processing, and production of hepatic VLDL, and there are evidences in literature suggesting that *APOE* polymorphisms affect the stability of ApoB-100 or ApoB truncations ^19^. We speculated that the identified variant in *APOE* could have a role in disease pathogenesis, although the underlying mechanisms are not yet identified. In one patient (#39) presenting with increased LDL levels (247 mg/dL) and SLD, we identified a rare heterozygous variant in *APOB* gene (NM_000384: p.D1113H), already reported to be possibly associated with autosomal dominant Familial hypercholesterolemia type 2, characterized by severely elevated LDL cholesterol levels due to reduced clearance of ApoB caused by gain-of-function mutations ^20^. Of note, this patient had a high SLD-PRS score that could contribute to hepatic manifestations. In another patients (#40) presenting with high LDL levels, we identified a rare heterozygous variant in *GBE1* gene (NM_000158.4: p.Y329C) associated with Glycogen storage disease IV and fat accumulation ^21^; the same variant was associated in UKBB with chronic pancreatitis.

**Polygenic predisposition to steatotic liver disease**

Genetic predisposition to SLD, as captured by SLD-PRS that evaluate the rare combinations of common variants in genes associated with hepatic lipid metabolism (*PNPLA3-TM6SF2-GCKR-MBOAT7*, adjusted for a protective variant in *HSD17B13*), contributes to severe or early onset disease phenotypes. In the overall cohort, a genetic predisposition to SLD was detected in 17 out of 49 patients (35%). In five patients, the associated presence of pathogenic rare variants allowed to establish a genetic diagnosis of Mendelian disorders. In nine cases, the high SLD-PRS was associated to rare VUS, and their combination could contribute to clinical phenotype. Three patients (#41, #42, #43) had a high SLD-PRS value without associated rare variants. They presented with severe SLD, evolved to advanced fibrosis in the first two subjects (LSM 14.6 kPa and 22.2 kPa, respectively). All were homozygous for the *PNPLA3* p.I148M common variant.

**SUPPLEMENTARY REFERENCES**

1. Pelusi S, Ronzoni L, Malvestiti F, et al Clinical exome sequencing for diagnosing severe cryptogenic liver disease in adults: A case series. Liver Int. 2022;42(4):864-870.
2. Szustakowski JD, Balasubramanian S, Kvikstad E, et al. Advancing human genetics research and drug discovery through exome sequencing of the UK Biobank. Nat Genet. 2021 Jul;53(7):942-948.
3. Youssefian L, Vahidnezhad H, Saeidian AH, et al. Inherited non-alcoholic fatty liver disease and dyslipidemia due to monoallelic *ABHD5* mutations. J Hepatol. 2019;71(2):366-370.
4. Yang A, Mottillo EP, Mladenovic-Lucas L, Zhou L, Granneman JG. Dynamic interactions of *ABHD5* with *PNPLA3* regulate triacylglycerol metabolism in brown adipocytes. Nat Metab. 2019;1(5):560-569.
5. Dixon PH, Sambrotta M, Chambers J, et al. An expanded role for heterozygous mutations of *ABCB4, ABCB11, ATP8B1, ABCC2* and *TJP2* in intrahepatic cholestasis of pregnancy. Sci Rep. 2017;7(1):11823.
6. Jüngst C, Justinger C, Fischer J, Berg T, Lammert F. Common *ABCB4* and *ABCB11* Genotypes Are Associated with Idiopathic Chronic Cholestasis in Adults. Dig Dis. 2022;40(4):489-496.
7. Bull LN, Thompson RJ. Progressive Familial Intrahepatic Cholestasis. Clin Liver Dis. 2018;22(4):657-669.
8. Nayagam JS, Foskett P, Strautnieks S, et al. Clinical phenotype of adult-onset liver disease in patients with variants in *ABCB4, ABCB11*, and *ATP8B1*. Hepatol Commun. 202;6(10):2654-2664.
9. Pelusi S, Baselli G, Pietrelli A, et al. Rare Pathogenic Variants Predispose to Hepatocellular Carcinoma in Nonalcoholic Fatty Liver Disease. Sci Rep. 2019;9(1):3682.
10. Zheng M, Hakim A, Konkwo C, et al. Advancing diagnosis and management of liver disease in adults through exome sequencing. EBioMedicine. 2023;95:104747.
11. Vilarinho S, Ajmera V, Zheng M, Loomba R. Emerging Role of Genomic Analysis in Clinical Evaluation of Lean Individuals With NAFLD. Hepatology. 2021;74(4):2241-2250.
12. Balcar L, Scheiner B, Urheu M, et al. Alpha-1 antitrypsin Pi∗Z allele is an independent risk factor for liver transplantation and death in patients with advanced chronic liver disease. JHEP Rep. 2022;4(11):100562.
13. Cordell HJ, Han Y, Mells GF, et al. International genome-wide meta-analysis identifies new primary biliary cirrhosis risk loci and targetable pathogenic pathways. Nat Commun. 2015;6:8019.
14. Benyamin B, McRae AF, Zhu G, et al. Variants in TF and HFE explain approximately 40% of genetic variation in serum-transferrin levels. Am J Hum Genet. 2009;84(1):60-5.
15. Corradini E, Buzzetti E, Dongiovanni P, et al. Ceruloplasmin gene variants are associated with hyperferritinemia and increased liver iron in patients with NAFLD. J Hepatol. 2021;75(3):506-513.
16. Guldiken N, Hamesch K, Schuller SM, et al. Mild Iron Overload as Seen in Individuals Homozygous for the Alpha-1 Antitrypsin Pi*Z Variant Does Not Promote Liver Fibrogenesis in HFE Knockout Mice. Cells. 2019;8(11):1415.
17. Rametta R, Dongiovanni P, Baselli GA, et al. Impact of natural neuromedin-B receptor variants on iron metabolism. Am J Hematol. 2020;95(2):167-177.
18. Pronicka E, Węglewska-Jurkiewicz A, Taybert J, et al. Post mortem identification of deoxyguanosine kinase (DGUOK) gene mutations combined with impaired glucose homeostasis and iron overload features in four infants with severe progressive liver failure. J Appl Genet. 201;52(1):61-6.
19. Groenewegen WA, Krul ES, Averna MR, Pulai J, Schonfeld G. Dysbetalipoproteinemia in a kindred with hypobetalipoproteinemia due to mutations in the genes for ApoB (ApoB-70.5) and ApoE (ApoE2). Arterioscler Thromb. 1994;14(11):1695-704.
20. Abifadel M, Boileau C. Genetic and molecular architecture of familial hypercholesterolemia. J Intern Med. 2023;293(2):144-165.
21. Yang L, Sun Z, Li J, et al. Genetic Variants of glycogen metabolism genes were associated with liver PDFF without increasing NAFLD risk. Front Genet. 2022;13:830445.

**SUPPLEMENTARY TABLES**

**Table S1**. Demographics and clinical information of each patient

BMI: body mass index; T2D: type 2 diabetes; HDL_ high density lipoprotein; LDL: low density lipoprotein; TGL: triglycerides; AST: aspartate transaminase; ALT: alanine aminotransferase; GGT: gamma-glutamyl transferase; HbA1c: glycated hemoglobin A1c; PLTs: platelets; LSM: liver stiffness measurement

**Table S2**. Clinical features of the hypercholesterolemia and HBL subgroups

Data are shown as N (%), or median [IQR], when appropriate. M: male; BMI: body mass index; T2D: type 2 diabetes; LSM: liver stiffness measurement; AST: aspartate transaminase; ALT: alanine aminotransferase; GGT: gamma-glutamyl transferase; LDL: low density lipoprotein. na: not available. *P values were calculated among pairs through Kruskal-Wallis test for continuous variables (non-normality assumed) and Fisher test for categorical variables

**Table S3**. Genetic information of patients for whom a genetic diagnosis was not established

° Allele frequency in East Asian ancestry

**Table S4**. Variants significantly associated with liver traits in UKBB

Abbreviations: ALT: alanine aminotransferase; LDL: low density lipoprotein; ALP: alkaline phosphatase; PDFF: proton density fat fraction

**SUPPLEMENTARY FIGURE**

**Figure S1**. Example of a clinical report.

**SUPPLEMENTARY FILE**

**File S1.** R code for clinical report generation
