# Supplementary material for: Diagnostic Uptake of Targeted Sequencing in Adults With Steatotic Liver Disease and a Suspected Genetic Contribution
Source: Liver Int. 2025 Feb 13;45(3):e70010. doi: 10.1111/liv.70010 (PMC11822878; doi:10.1111/liv.70010)
Supplement: Supplementary file 4 — Table S2. [file LIV-45-0-s004.docx]

**Table S2**. Clinical features of the hypercholesterolemia and HBL subgroups

|  | SLD & Hypercholesterolemia | SLD & HBL | p value |
| --- | --- | --- | --- |
|  | (n=3) | (n=3) |  |
| Age (years) | 44 [39;49] | 29 [20;30] | 0.049 |
| Sex, M | 3 (100) | 3 (100) | na |
| Caucasian, yes | 2 (67) | 3 (100) | 1 |
| Family history, yes | 2 (67) | 3 (100) | 1 |
| BMI (kg/m2) | 27 [24;30] | 25 [25;29] | 0.8 |
| T2D, yes | 0 | 0 | na |
| Liver fibrosis, yes | 0 | 1 (33) | 1 |
| Cirrhosis, yes | 0 | 0 | na |
| LSM (Kpa) | 5.9 [4.5;5.9] | 6.6 [4.5;9.9] | 0.3 |
| AST (IU/L) | 19 [18;30] | 35 [20;36] | 0.1 |
| ALT (IU/L) | 28 [26;28] | 53 [17;69] | 0.5 |
| GGT (IU/L) | 32 [28;48] | 16 [14;71] | 0.5 |
| LDL (mg/dL) | 247 [213;252] | 24 [15;45] | 0.003 |
| Triglycerides (mg/dL) | 122 [106;182] | 38 [36;63] | 0.04 |
| Ferritin (ng/mL) | 67 [48;300] | 56 [44;306] | 0.8 |

Data are shown as N (%), or median [IQR], when appropriate. M: male; BMI: body mass index; T2D: type 2 diabetes; LSM: liver stiffness measurement; AST: aspartate transaminase; ALT: alanine aminotransferase; GGT: gamma-glutamyl transferase; LDL: low density lipoprotein. na: not available. *P values were calculated among pairs through Kruskal-Wallis test for continuous variables (non-normality assumed) and Fisher test for categorical variables
